# Influence of Magnetic Field on the Distribution of the Ferromagnetic Component in Centrifugally Cast Ceramic-Metal Gradient Composites

**DOI:** 10.3390/ma14040955

**Published:** 2021-02-18

**Authors:** Justyna Zygmuntowicz, Marcin Wachowski, Dominika Zielant, Waldemar Kaszuwara

**Affiliations:** 1 Faculty of Materials Science and Engineering, Warsaw University of Technology, 141 Woloska Str., 02-507 Warsaw, Poland; dominika.zielant@gmail.com (D.Z.); Waldemar.Kaszuwara@pw.edu.pl (W.K.); 2 Faculty of Mechanical Engineering, Military University of Technology, 2 gen. S. Kaliskiego St., 00-908 Warsaw, Poland; marcin.wachowski@wat.edu.pl

**Keywords:** magnetic field, centrifugal slip casting, ferromagnetic component

## Abstract

The main aim of the investigation was to determine the impact of the content of nickel and the content of slurry on the nature of the microstructure and physical properties of the final products. In the study, six types of slurries were examined and prepared, differing in both the amounts of content of Ni metallic phase particles (5 vol.%, 10 vol.%, and 20 vol.%) and the amount of content of solid content in the prepared slurries (35 vol.%, and, 50 vol.%). The centrifugal slip casting (CSC) method in a magnetic field was used to fabricate the composites. This technique allowed the production of high-density ZrO_2_-Ni composites after sintering. Composites containing 50 vol.% of the solid content were characterized by a relative density equal to 99%. Applying the magnetic field allows controlling the distribution of the ferromagnetic phase (Ni) in the ceramic matrix (ZrO_2_). Based on the results obtained, it was found that the nature of the composites obtained is influenced by the rheological properties of the slurries, depending on their composition. The applicability of the CSC in the magnetic field technique for the production of the composite is characterized by a gradient in the distribution of components on the longitudinal section and has been proved. Based on the obtained results, a model for shaping the microstructure of composites with a longitudinal section was proposed. This work enabled a better understanding of creating microstructures in materials fabricated by centrifugal slip casting in a magnetic field.

## 1. Introduction

The constant development of many fields of technology generates the need for improving available materials. Ceramic-metal composites with unique properties stay particularly popular. It should be emphasized that nowadays, many applications require the use of a material whose physicochemical properties change in the chosen direction. In response to this demand, gradient materials have been developed, characterized by a constant change in the concentration of components in a given order [1,2,3,4,5,6,7,8,9,10]. In functionally graded materials (FGM), the lack of abrupt changes in the microstructure may avoid the appearance of defects on the border of the separation of individual phases. One of the fundamental problems of forming gradient composites from a ceramic-metal system is controlling particle distribution in the volume of the materials. The nature of the microstructure depends on both the manufacturing process parameters and the composition of the raw materials. One of the fundamental methods of obtaining gradient composites is classical powder metallurgy. Shaping the product using this technique boils down to preparing appropriate powder mixtures and backfilling of the matrix. The formed product is compacted by pressing and then sintered. The powder metallurgy technique allows shaping a microstructure characterized by a variable distribution of metallic and/or ceramic particles on the cross-section or longitudinal section of the produced composites [11,12,13,14,15,16,17,18]. The gradient structure of composites can also be obtained using so-called “wet” methods, in which slurries are used [19,20]. The slurry is called a suspension, which may contain, e.g., ceramic powders and metallic powders, solvents, binders, as well as the addition of surfactants. Among these methods, stand out among others, tape casting [21,22,23,24], slip casting [25,26], as well as centrifugal slip casting [27,28,29].

The demand for composite materials is continuously increasing. New methods of forming gradient composites are still being sought that would allow for the production of high-quality materials with a planned microstructure. To enable series production, it is necessary to develop a technique leading to the production of materials with a repeatable structure while considering the economic and ecological aspects of the method of forming composite products. Therefore, this paper presents a process of obtaining gradient ZrO_2_-Ni composites by centrifugal casting in a magnetic field. The focus was on determining the effect of slurry composition on metals distribution with ferromagnetic (Ni) properties in the ZrO_2_ matrix. The presence of a magnetic field allowed us to obtain the other structure than in the case of composite composites using the technique of centrifugal casting of ceramic-metallic suspension without a magnetic field.

The initial studies about the Al_2_O_3_-Ni system provided a theoretical background for employing centrifugal slip casting in the magnetic field for fabricating and composites of the final product in the form of hollow tubes [30,31]. The results indicated that the magnetic field could influence the distribution of metal particles in the ceramic matrix [30,31]. Authors observed that simultaneous action of centrifugal force and strong magnetic field results in obtaining a characteristic material structure with nickel particle chains. The content of the solid phase in the slurry affects its viscosity, which, along with its increase, changes the character of the distribution of the metallic chains in the volume of the material. Authors in article [30] purpose a model for the formation of such a structure. Therefore, this work is a continuation of previous research conducted by the team on the production of ceramic-metal composites obtained by centrifugal casting using a magnetic field. In these investigations, we attach great importance to characterize the distribution of the particles in the longitudinal section of the fabricated composites. Our study is fundamentally different from previous research since, in our previous studies, we examined the distribution of the particles mainly on the cross-section samples. Furthermore, we believe that it will be interesting to use another ceramic matrix than the Al_2_O_3_. Therefore, in these investigations, we used ZrO_2_. We believe it will help us to better understand the influence of the magnetic field on the distribution of ferromagnetic particles in a ceramic matrix composite. The research described in this publication aims to determine the influence of magnetic field on the distribution of the ferromagnetic component in centrifugally cast ceramic-metal gradient composites.

## 2. Materials and Methods

**Figure 1:**
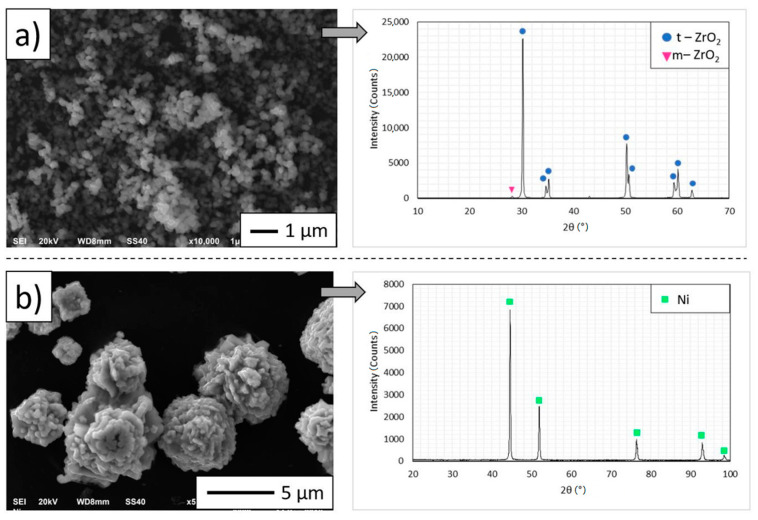
Scanning electron micrograph and XRD pattern of raw materials: (**a**) ZrO_2_, (**b**) Ni.

The ceramic powder used in the research was ZrO_2_ TZ-3YS-E powder (Tosoh Co., Tokyo, Japan) with an average particle size of 0.10 ± 0.02 µm and density 5.88 g·cm^−3^. The densities were measured by helium pycnometer AccuPyc II 1340 (Micromeritics Instruments, Norcross, GA, USA). The ZrO_2_ powder was stabilized by 3 mol% Y_2_O_3_. However, it was used as a metallic Ni powder (Createc, Stalowa Wola, Poland) with an average size of mean particle size in the range of 3 to 7 μm and density 8.91 g·cm^−3^. The properties of the raw powders are shown in Table 1. The specific surface area was specified by the Brunauer-Emmett-Teller adsorption isotherm (BET) method with the aid of ASAP 2020 V3. 01H (Micromeritics Instruments) gives the most accurate outcomes among other accessible standard methods. The results reveal that the specific surface area for ZrO_2_ powder was 6.7 ± 0.09 m^2^·g^−1^, while for Ni powder, it is 0.31 ± 0.01 m^2^·g^−1^, respectively. The scanning electron micrographs and XRD analysis of the morphology of the raw materials were shown in Figure 1. SEM micrograph (Figure 1a) showing the characteristic spherical morphology of ZrO_2_ nano powder particles. The observation of ceramic powder exhibits that zirconia is characterized by the tendency to be firmly agglomerated. While the direct observation of the metal powder allowed us to find that Ni powder shows an irregular shape (Figure 1b). Obtained results allowed us to observe that in the powder, XRD analysis showed no other phases except for the nickel phase. While, the x-ray investigation indicates that the structure of zirconia oxide is characterized by two phases: monoclinic (m-ZrO_2_, characteristic card PDF# 04-010-6452) and tetragonal (t-ZrO_2_, characteristic card PDF# 04-055-4504). The presence of two phases is the effect of the partial stabilization by 3 mol.% Y_2_O_3_. The result of the experiment reveals that in the raw monolith ZrO_2_ powder, 35.3 wt.% of monoclinic zirconia and 64.7 wt.% of tetragonal zirconia were determined. The obtained measurements of the phase composition of the powder showed that ZrO_2_ is not fully tetragonal and the content of the monoclinic phase with lower density lowers the average density of the mixture of ZrO_2_ powder. In addition, it should be borne in mind that the differences arising from the density measured by the pycnometer method, and the density given by the manufacturer, may be due to a measurement error, which is associated with insufficient desorption of raw powders before measurement. In the case where if it is not possible to perform full desorption, the volume is overstated and, therefore, the density given by the device is lower.

**Figure 2:**
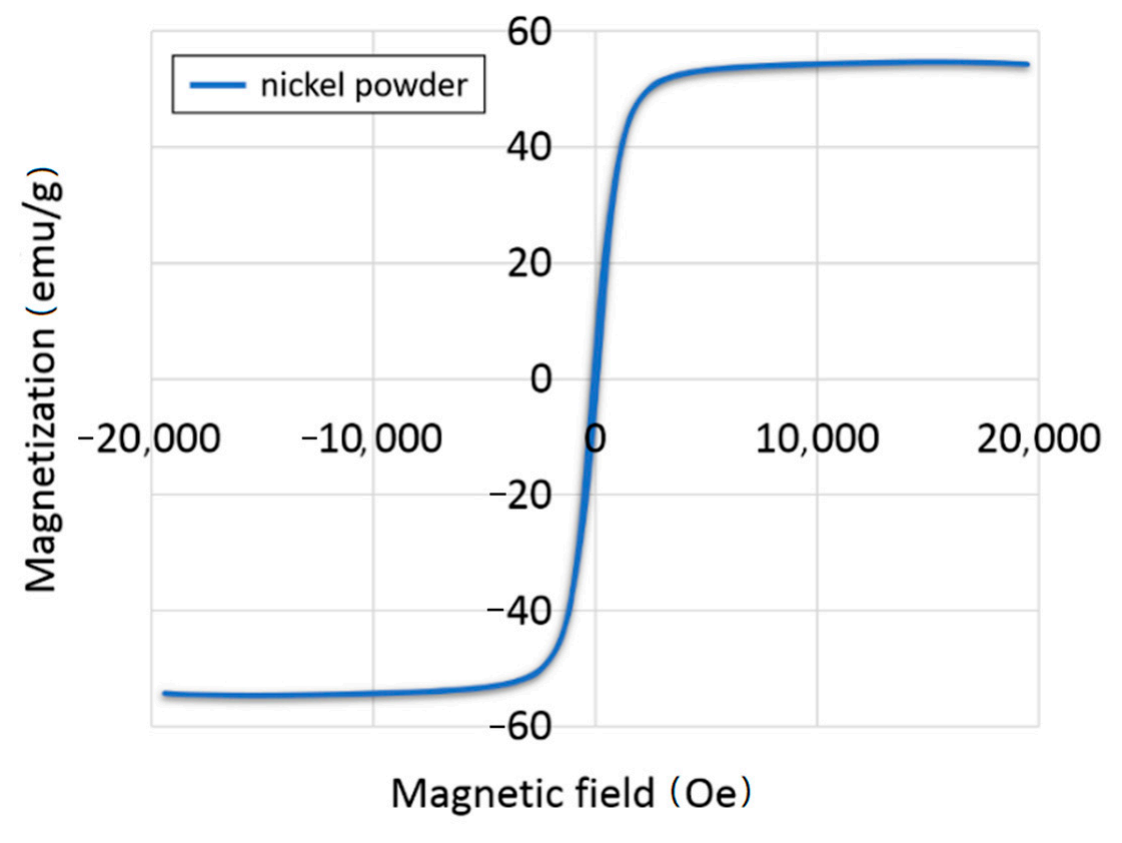
Ferroelectric hysteresis loop of magnetic nickel powder.

The magnetic properties of the metal powder were determined using the device employing the vibration sample magnetometer (Lake Shore Model 7404 VSM, Lake Shore Cryotronics, Westerville, OH, USA). The experiment was done at room temperature in a varying magnetic field. Based on the obtained hysteresis loop (Figure 2), remanence magnetization (M_r_), saturation magnetization (M_s_), and coercivity (H_ci_) were specified. The measurement done with the vibrating sample magnetometer (VSM) allowed us to observe the shape of the hysteresis loop, which is shown in Figure 2, revealed evidence of the ferromagnetic character of the Ni powder. The obtained results demonstrated that the ability of a Ni powder to obtain magnetization is equal to 2.6494 emu·g^−1^. Based on the hysteresis loops, it can be observed that Ni was characterized by coercively (H_ci_) of 60.174 Oe. In addition, it was noted that the value of saturation magnetization was 54.715 emu·g^−1^.

As part of the work, composites from the ZrO_2_-Ni system were produced by the centrifugal slip casting in a magnetic field. For this purpose, six types of slurry were prepared, differing in both the amounts of content of Ni metallic phase particles (5 vol.%, 10 vol.%, and 20 vol.%) and the amount of content of solid content in the prepared suspension (35 vol.% and 50 vol.%). Citric acid, CA (99.5%, Sigma-Aldrich, St. Louis, MO, USA), and diammonium hydrocitrate, DAC (Avantor Performance Materials Poland S.A. (formerly POCH S.A.), Gliwice, Poland) were used as dispersing agents for the particles in the suspensions. In the experiment, water was used as the solvent. The choice of this solvent was dictated by the fact that it has no toxic properties and is cheap. The concentrations of all components in the slurries are listed in Table 2.

**Figure 3:**
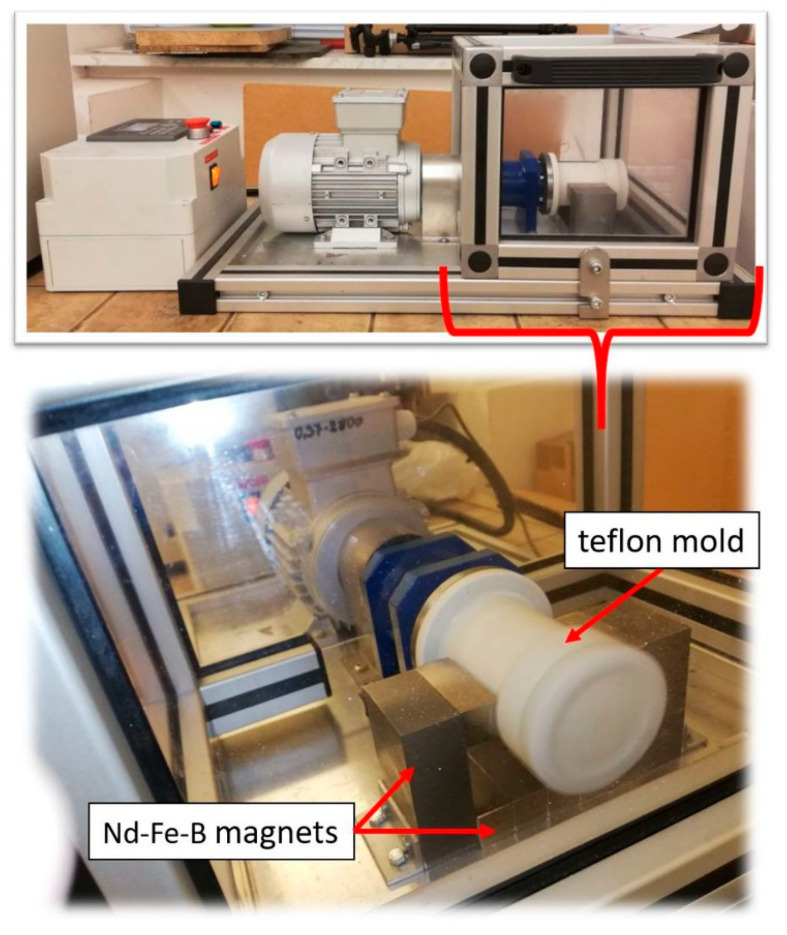
Stand for the production of composites by the method centrifugal slip casting in the magnetic field.

The first stage in the production of composites was weighing the right amount of individual components. The prepared slurries were placed in a Thinky ARE-250 high-speed homogenizer (Thinky Corporation, Tokyo, Japan), where the mixing and degassing processes of the suspension occurred alternately. The slurry thus prepared was poured into a porous gypsum mold, which was placed in a centrifuge housing. The casting process was carried out in a horizontal system (the centrifuge axis was horizontal) to eliminate the effect of gravitational sedimentation of the metallic powder. To obtain an external magnetic field in the system used for forming composites, N38 neodymium magnets imported by ENES magnets were installed. Seven Nd-Fe-B magnets with dimensions of 5 mm × 2 mm × 20 mm have been arranged to generate a magnetic field around the gap in which the mold with the prepared slurry was placed. The device for casting composites is shown in Figure 3. The casting process was carried out at 3000 rpm for 130 min at room temperature. The resulting green body composites were dried for 48 h at 40 °C and then sintered in a H_2_/N_2_ reducing atmosphere. The sintering process parameters were selected on the basis of previous experimental studies [30,31]. Sintering of the composites was carried out in the following stages: heating up to 120 °C, heating speed: 5 °C·min^−1^, heating in the temperature range 120–750 °C, heating speed: 2 °C·min^−1^, heating in the temperature range of 750–1400 °C, heating speed: 5 °C·min^−1^, dwelling at 1450 °C, the dwell time: 2 h, cooling speed: 5 °C·min^−1^. As a result of the technological process, composites of cylindrical shape were obtained. The samples produced had a length of about 45 mm and a diameter of about 16 mm. In this study, it was assumed that centrifugal casting in a magnetic field should lead to a variable distribution of the metallic phase and the ceramic phase.

In this article, several studies have been carried out to characterize the slurries used to produce composites, magnetic fields, and composites.

The rheological properties of the suspensions were examined in a rheometer Anton Paar MCR102 (Anton Paar GmbH, Graz, Austria). The study of dynamic viscosity and shear stress was set by rotating the spindle in the speed range 1.3–260–1.3 rotations per minute, where buoyancy was electronically controlled. The rheological investigations were taken in an air-conditioned lab at 23 °C.

**Figure 4:**
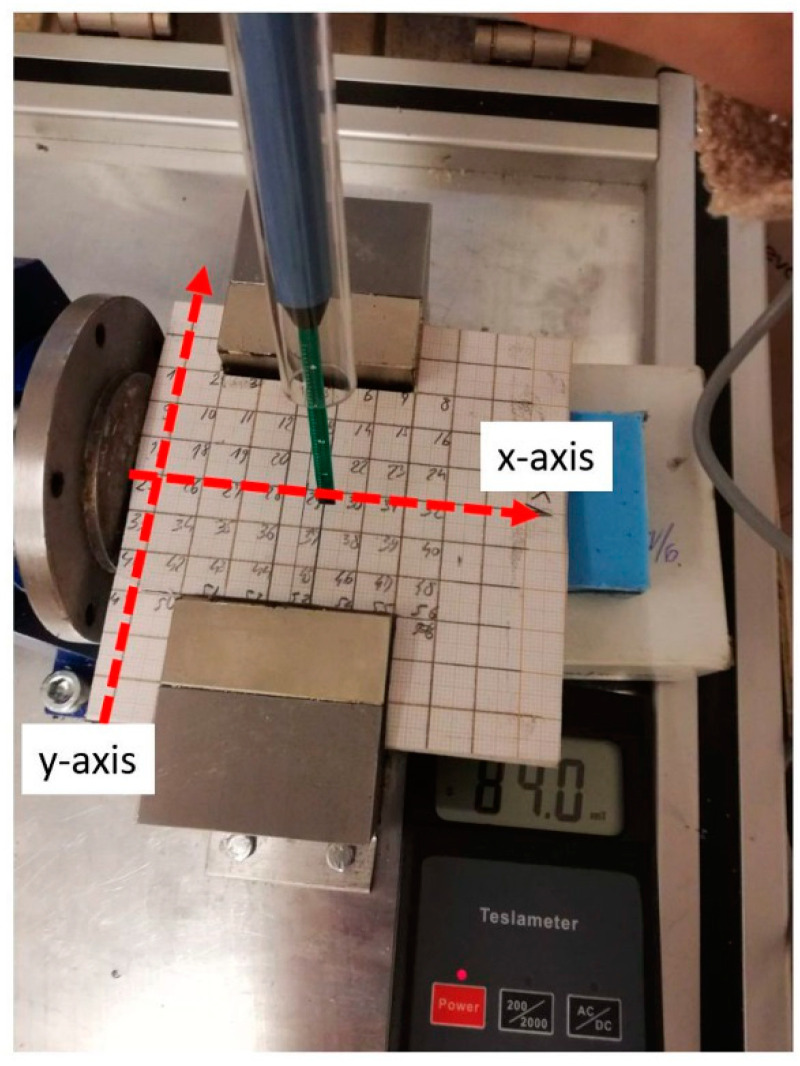
Location of measuring points for the magnetic field.

To analyze the distribution of the magnetic field strength generated by the magnets used, magnetic strength measurements were carried out. The longitudinal field, whose force line should be perpendicular to the centrifuge rotation axis (*y*-axis), and the transverse field, whose force lines should be parallel to the centrifuge axis (*x*-axis), were measured. The location of the measuring points is shown in Figure 4. The tests were carried out using the LES-641H teslometer ENES Magnesy (ENES Magnesy, Warsaw, Poland), equipped with a probe with a hallotron sensor. 

Macroscopic observations constituted the first stage of characterization of the fabricated composites. The tests were carried out directly after the centrifugation process, as well as after sintering. Detailed observation of the microstructure of the produced samples was made using a digital camera and a Nikon ECLIPSE LV150N light microscope (Tokyo, Japan), using the contrast resulting from the natural differences in light absorption for different phases. Before the test, the samples were ground and polishing. For the observation of the longitudinal section surface of the composites, the samples were cut in a direction parallel to the length of the composite. Then, a series of photos of the longitudinal section surface were taken, which were then assembled into a panorama. Taken pictures of the panorama sections along the axis of the sample. Observations were carried out at a magnification of 5× and 10×. 

The density of ZrO_2_-Ni composites after sintering was determined by hydrostatic weighing in accordance with ISO 18754: 2013 (EN). The method is based on Archimedes principle by which it is possible to determine the volume of the sample immersed in the liquid of known density without specifying its geometrical dimensions. The measurement is based on determining the mass of the dry sample, followed by its saturation in water and reweighed—in air and in water. The theoretical density was determined based on the density of the powders specified by the manufacturers: ZrO_2_ = 5.88 g·cm^−3^ and Ni = 8.9 g·cm^−3^.

X-ray analysis is a versatile technique that is often used in materials science. XRD studies provide a range of information regarding the structure and phase composition of crystalline materials. It allows us to explore, among others, crystallite size, unit cell parameters, crystallographic texture and enables quantitative and qualitative analysis of phase composition. The phase composition of the used powders and manufactured ZrO_2_-Ni composites after sintering was determined on the basis of measurements done on a Rigaku MiniFlex II X-ray diffractometer (Rigaku Corporatio, Tokyo, Japan). CuK_α_ radiation with a wavelength λ = 1.54178 Å was used. The tests were carried out at 30 kV voltage and 15 mA current, in the 10°–70° and 20°–100° angle range for powders and composites, respectively, at 0.02°, during the counting of 2 s.

## 3. Results and Discussion

**Figure 5:**
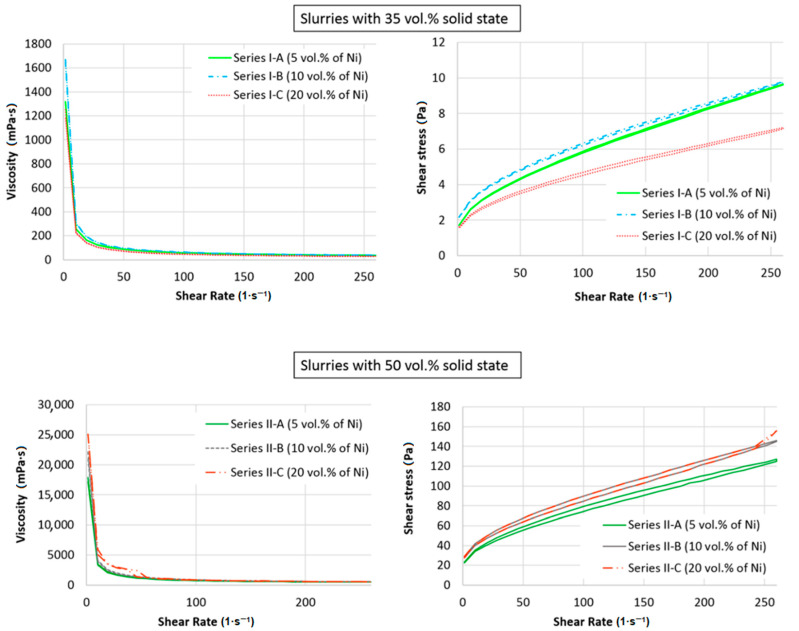
Viscosity curves and flow curves of the ZrO_2_-Ni composite slurry.

A centrifugal slip casting with/without magnetic field method needs the suitable fluidity of suspension. The viscosity and thus the fluidity of the suspension is performed by many factors that determine its properties. Therefore, it is important that the first stage of research is to determine the rheological properties of the prepared slurries. Figure 5 presents the collected results of rheological measurements in the form of viscosity curves and flow curves for the slurry produced in the experiment. The obtained curves for all tested suspensions indicate the non-Newtonian character of the tested slurries. The results indicated that the slurries’ viscosity decreases as the shear rate increases, which means that these are shear thinning liquids. The phenomenon of shear thinning can be explained by the presence of agglomerates of particles in the suspension that cause an apparent increase in the volume concentration of the solid phase leading to an increase in viscosity. As a result of stresses during the suspension flow, the agglomerates are broken up, which in turn leads to the release of part of the liquid phase trapped between the particles, an apparent decrease in the concentration of the solid phase, and as a consequence, a decrease in viscosity. Based on Figure 5 with the flow curves, τ = f (γ) created under conditions of successively increasing and decreasing shear rate, a characteristic course with a hysteresis loop was observed (Figure 5) in all slurries examined. Shear stress values increase as the shear rate increases. It was found that when the shear rate decreases, the stress decreases again, and the curve formed at decreasing speed lies under the curve formed at an increasing shear rate. These observations indicate the thixotropic properties of all suspensions tested. As previously noted, the viscosity of ceramic slurries decreases as the shear rate increases due to the gradual destruction of the structure. The highest viscosity for all suspensions used for testing was observed at a shear rate of γ = 1.3 (1·s^−1^), while the lowest viscosity values were obtained at a shear rate of γ = 260 (1·s^−1^). The obtained viscosity and stress values of the tested slurry at maximum and minimum shear rate are presented in Table 3. The obtained results of measurements of the rheological properties of the analysed slurries indicate that their viscosity decreases with increasing shear rate, and therefore they can be classified as non-Newtonian liquids diluted with shear. It was found that the suspensions produced had a clear flow limit. Furthermore, based on the results obtained, it can be stated that the higher the solids content in the slurry, the higher the viscosity. Moreover, the experiments demonstrated that the content of Ni particles in the suspension also affects the rheological properties. This is also correlated with the increase in mass solids content.

**Figure 6:**
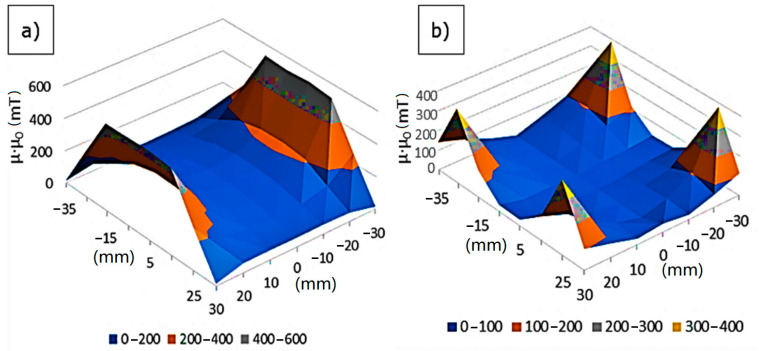
Magnetic field strength distribution in a system for casting mold mass: (**a**) longitudinal measurement, (**b**) cross magnetic field measurement.

Measurements of the longitudinal and cross magnetic field intensity allowed to determine its changes in the horizontal plane containing the sample’s rotation axis. Based on the maps of the distribution of magnetic field strength values measured in the longitudinal direction (Figure 6a), it was determined that it reaches the highest value on the magnet’s surface in the magnetic system axis (at the intersection of the diagonals of the magnet’s front wall) and is about 540 mT. Direct measurements have shown that the magnetic field strength along the *x*-axis decreases slightly towards the side surface of the magnet and then decreases sharply. Along the *x*-axis, i.e., along the axis of rotation of the mold, it was observed that these values decrease as the distance from the surface of the magnet, and the lowest value was observed in half the distance between the magnets (in the axis of the centrifuge)—approx. 125 mT. Measurements of the cross magnetic field strength (Figure 6b) indicated that the maximum magnetic field value occurs at the corners of the area under study. The measurements showed that the simple magnetic system used does not provide a homogeneous magnetic field.

**Figure 7:**
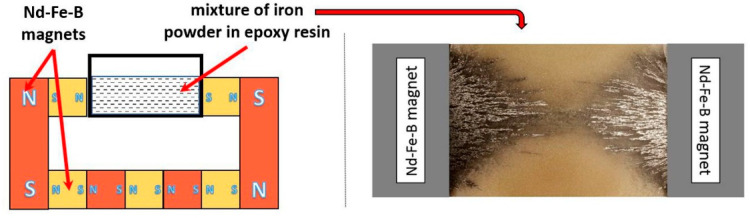
Schema of the located magnets in equipment with fabricated materials and a photo showing the sample with the distribution of iron powder particles.

An attempt was made to initially determine the effect of magnetic field distribution on the distribution of ferromagnetic powder particles in the gap between the magnets. For this purpose, a container was placed in the gap, into which a mixture containing 10 vol.% of iron powder in Epidian 5 epoxy resin and Z-1 hardener was introduced (Figure 7). After the resin cured, the surface of the obtained specimen was levelled and its image is shown in Figure 7.

Based on Figure 7, it was found that the magnetic field distribution determines the distribution of ferromagnetic powder particles in the gap between the magnets. The powder is concentrated at the near the surface of the magnets, where the highest magnetic field strengths were measured (Figure 7). A large gradient of this magnitude along the *y*-axis means that as the distance from the magnet surface increases, the powder content decreases rapidly. At the same time, the gradient of the magnetic force along the *x*-axis causes the powder to concentrate in the region of the center of the magnet (axis of rotation). The powder particles form chains lying along the magnetic field force lines. In the central part of the gap, these lines are perpendicular to the axis of rotation (*x*-axis). In the lateral part of the gap (around the lateral edges of the magnets), these lines diverge in the *y*-axis direction. This is due to significant values of the magnetic field in the corners of the gap. The described experiment allowed us to determine the initial distribution of ferromagnetic powder in the magnetic system. This facilitated the interpretation of microstructure images of compressed centrifugally cast magnetic fields. 

**Figure 8:**
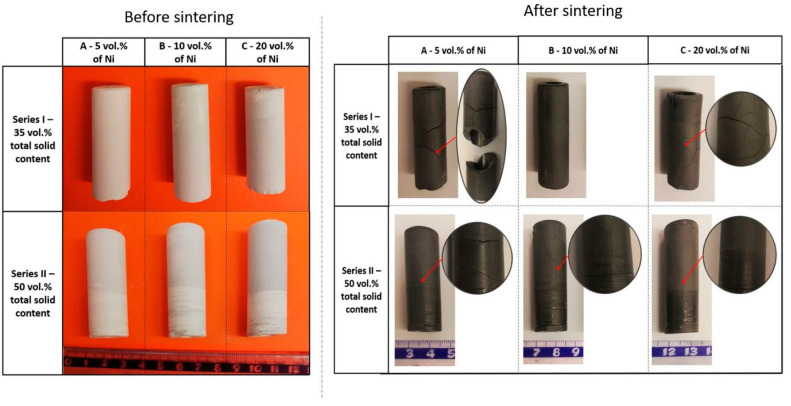
Photos of specimens before and after sintering.

Figure 8 shows example pictures of the green body and samples after the sintering process obtained by centrifugal slip casting with the magnetic field. From the macroscopic observation, it may be concluded that specimens before the sintering process characterize no cracks or delamination on the surface. Unfortunately, after the sintering process, part of the produced composites had cracked. Based on the observation of Series I-A and Series I-C samples, it was noticed that they had numerous cracks. It can be assumed that the cracks were caused by a large difference in the thermal expansion coefficients of Ni and ZrO_2_. The most satisfactory results were obtained for the Series I-B composite. It was found that the samples from Series I-B were characterized by the absence of cracks and delamination on the surface after the sintering process. In addition, it was noted that the photographs of Series II-A and Series II-B samples show the presence of many cracks that occur only on the outer surface of the samples. In these samples, no cracks along the transverse surfaces were observed in contrast to samples from the Series II-C sample, where surface cracks were noted. 

**Figure 9:**
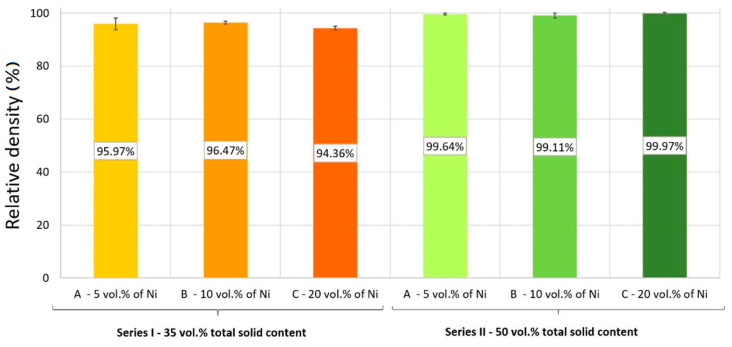
Values of the relative density of produced composites.

**Figure 10:**
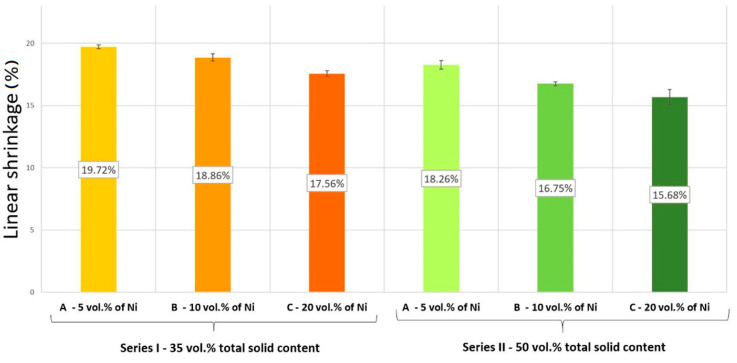
Values of linear shrinkage of the produced composites.

The results of relative density and linear shrinkage were shown in Figure 9 and Figure 10. The results obtained provided information that the relative densities of Series I samples were in the range of 94–97%, and the values obtained for Series II samples were each value above 99%. It was found that the highest densification among Series I composites was obtained for a Series I-B (10 vol.% of Ni), equal to 96.47 ± 0.56%. While the minimum value of 94.36 ± 0.74% was recorded for Series I-C. The reason for obtaining the lowest density of the composite of Series I-B may be the presence of many cracks in the tested samples. It was noticed that with an increase in the metal content in the slurries used to obtain composites, the value of densification increased as well. The relative density of samples produced from suspensions with 50 vol.% of solid-phase exceeds 99%. It was noticed that in the case of Series II, the lowest value reached for Series II-B (99.11 ± 0.89%). Scientific knowledge and our previous investigations allow for the statement that as a result of centrifugal force during the process of forming composites, smaller ZrO_2_ particles may be filled the voids between Ni particles, which consequence allows composites with high densification. 

The results of linear shrinkage are presented in Figure 10. Prove that as the metallic phase increases, regardless of the type of series, a decrease in linear shrinkage was observed. It was found that the linear shrinkage of Series I samples was in the range of 17–20%. While the highest value (19.71 ± 0.16%) was recorded for the I-A Series composite, and the lowest value, 17.56 ± 0.22%, for the I-C Series. Based on the measurements, it was found that the linear shrinkage decreases with an increasing solids content in the slurry. In the case of Series II, the maximum value of 18.26 ± 0.34% was obtained for Series II-A, while the minimum value of 15.68 ± 0.62% for Series II-C.

**Figure 11:**
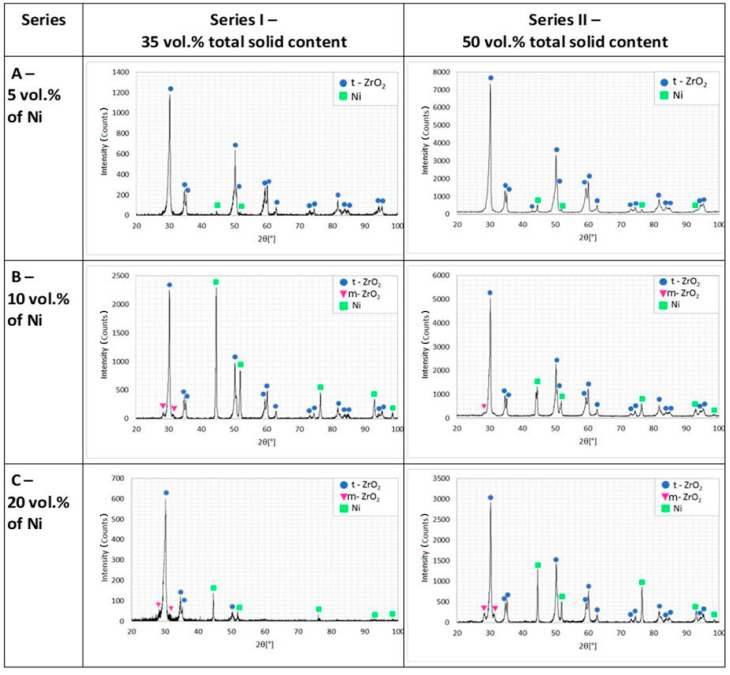
Diffractograms of composites after the sintering process.

Figure 11 has shown the results of X-ray diffraction analysis for each of the composites produced after sintering. The obtained results showed that in the case of samples with 5 vol.% of nickel content in the slurry used for the production of composites (Series I and Series II), only two phases Ni and t-ZrO_2_ were observed. While in the other composites, the presence of three phases was noted: Ni, t-ZrO_2_, and m-ZrO_2_. Perhaps the lack of m-ZrO_2_ phase in the sintered composites (Series I-A and Series II-A) could be the result of the preparation process of the composites from the observations and phase analysis, which was performed on the polish surface and applied loads and water promotes the reverse transformation (t → m). Both specialist literature data and our own research indicated that the additional stabilization of zirconia powder by Y_2_O_3_ allows gaining a high amount of t-ZrO_2_ after the sintering process. Specialist literature data show that the presence of the tetragonal phase is due to the fact that during the sintering process of ZrO_2_-Ni composites, the temperature of the monoclinic phase to tetragonal phase (over 1170 °C) changes was exceeded. 

**Figure 12:**
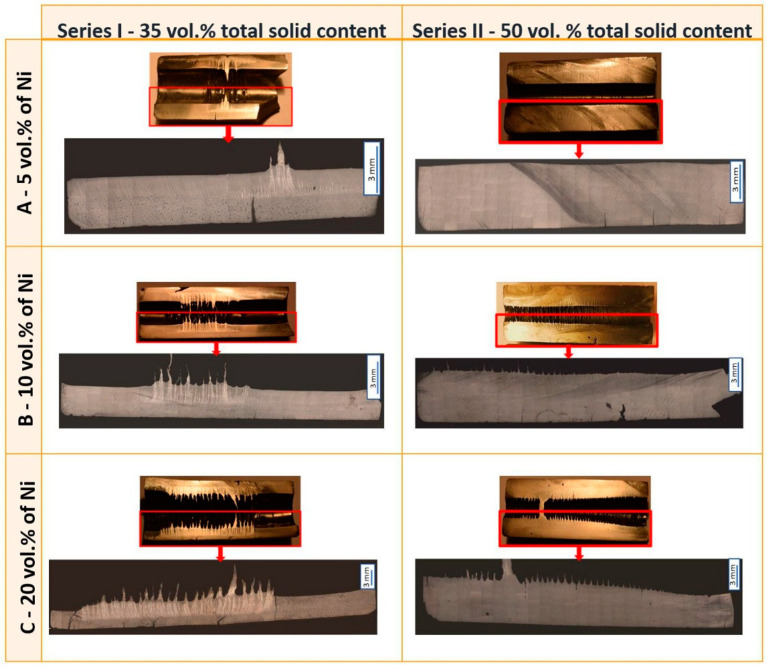
Example of the microstructure of the longitudinal section surface of the samples produced.

In the following part of the work, were presented macro photographs of a longitudinal section (taken with a digital camera) and a section showing the microscopic pictures of selected areas (Figure 12). In all samples, it can be seen that the metallic particles are concentrated in the central part of the sample. This phenomenon is caused by the inhomogeneity of the magnetic field in the applied system. Based on the observations, it can be concluded that increasing the volume of Ni content in a slurry width leads to an increase in the width of the zone rich in Ni. The nickel-rich zone in the case of all samples from Series I was characterized by the specific formation of the metallic phase in “tabs or moustache”. The obtained results demonstrated that increasing the solid content in the suspension increases its viscosity, which leads to a decrease in the displacement of Ni particles. From the above noted, it may be concluded that this results in a decrease in the tendency to “pull” Ni-rich areas to the inside the sample for samples with 50 vol.% of solids content (to form so-called “tabs”).

**Figure 13:**
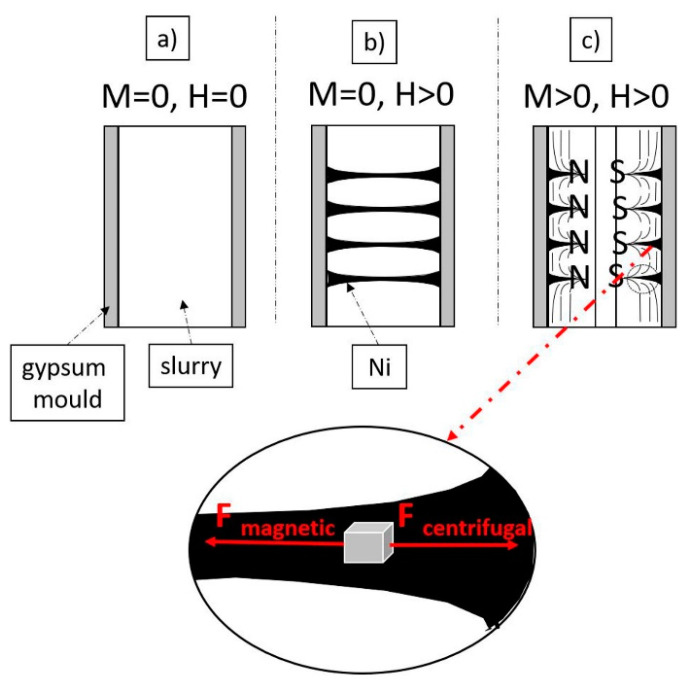
Diagram showing the model of the mechanism of formation of the microstructure of composites on the longitudinal section of materials obtained by centrifugal slip casting in a magnetic field (**a**) M = 0, H = 0; (**b**) M = 0, H > 0; (**c**) M > 0, H > 0.

On the basis of the obtained knowledge, a model of the mechanism of microstructure shaping on the longitudinal section of samples produced by the centrifugal slip casting in a magnetic field was proposed. A diagram of the proposed mechanism is shown in Figure 13. The obtained results lead to the conclusion that immediately after placing the slurry in the casting mold (under the conditions M = 0, H = 0), the suspension is affected by capillary forces coming from the gypsum mold. It causes the removal of water from the suspension, which leads to preliminary densification of the slurry. In the preliminary stage, the particles of the ferromagnetic metal are separated and evenly distributed in the suspension because in this case, we did not observe rotation (M = 0) and magnetic field (H = 0) (Figure 13a). In the next step, the use of a magnetic field (H > 0) leads to the magnetization of ferromagnetic Ni particles, as a result of which the metal particles join together and arrange along the magnetic field force lines (Figure 13b). In addition, adding the rotational movement of the mold (M > 0) causes the particles contained in the suspension to move in the direction consistent with the centrifugal force, i.e., towards the outer surface of the manufactured composite (Figure 13c). The acting centrifugal force causes “burst” of the formed chains of Ni. As a result of the manufacturing process, cylindrical samples with a complex microstructure are formed, in which nickel bands arranged in the direction of the magnetic field can be distinguished.

Based on the proposed mechanism, similar results can be anticipated in the future for other materials. The observations show that the proper selection of the proportion of Ni particles and the content of the solid phase in the slurry (determining its viscosity) may allow predictably shaping the distribution of ferromagnetic particles in the composite.

## 4. Conclusions

The results of this paper indicate that the method of centrifugal casting in a magnetic field enables to control the distribution of Ni particles in a ZrO_2_ matrix. It determines the correlation between the content of the solid phase and the participation of the ferromagnetic phase, and the microstructure of the produced composites. On the basis of the research, the following conclusions were formulated:

- The centrifugal slip casting in a magnetic field method allowed to produce high-density ZrO_2_-Ni composites after sintering.
- All tested slurries used to make the composites are shear-thinned suspensions. The measurements showed that the viscosity of the mass decreases with increasing shear rate. In addition, it was found that the suspensions used in the experiment had a clear flow point.
- Analysis of the phase composition of the tested materials showed that the use of a reducing atmosphere during the sintering process avoided the appearance of new phases in the composites. However, it was found that increasing the share of the metallic phase leads to an increase in the share of the m-ZrO_2_ phase at the expense of t-ZrO_2_.
- The use of an inhomogeneous magnetic field in the system leads to the aggregation of particles of nickel powder in the center part of the axis of the sample. The investigation indicated that the intensity of this phenomenon decreases with increasing viscosity and nickel content in the suspension used to obtain materials.
- The results demonstrated that the characteristic feature of the samples obtained is the presence of the so-called nickel-rich “tabs or moustache”. These are the areas where the arrangement of ferromagnetic particles along with the magnetic field force the line current above the inner surface of the sample. Based on the results obtained, a model for the formation of this type of microstructures was proposed.
- It was found that the use of slurry with a high content of solid-state and low content of nickel can prevent the appearance of areas so-called “tabs” nickel-rich in the material.

## Figures and Tables

**Table 1:** Properties of the raw powders.

Properties	ZrO_2_	Ni
Purity	99.99%	99.90%
Mean particle size from SEM investigation	0.09 ± 0.02 µm	4.3–5 μm
Mean particle size specified by the manufacturer	<0.1 μm	3–7 μm
The size of crystallites	380 Å	-
Density measured on helium pycnometer	5.88 g·cm^−3^	8.91 g·cm^−3^
Density specified by the manufacturer	6.05–6.10 g·cm^−3^	8.90 g·cm^−3^
Specific surface area (BET technique)	6.7 ± 0.09 m^2^·g^−1^	0.31 ± 0.01 m^2^·g^−1^
Melting point	2715 °C	1453 °C

**Table 2:** Composition of the slurries used in the investigations.

Type of Slurry	Total Solid Content	Nickel Powder	Citric Acid	Diammonium Hydrocitrate
	vol.%	vol.% with Respect to the Amount of the Solid Volume	wt.% with Respect to the Amount of the Ceramic Powder	wt.% with Respect to the Amount of the Ceramic Powder
Series I-A	35	5	0.1	0.3
Series I-B	35	10		
Series I-C	35	20		
Series II-A	50	5		
Series II-B	50	10		
Series II-C	50	20		

**Table 3:** Minimum and maximum values of viscosity and shear stress for the slurries tested.

Type of Slurry			Viscosity (mPa·s)		Shear Stress (Pa)	
			Shear Rate γ = 1.3 (1·s^−1^)	Shear Rate γ = 260 (1·s^−1^)	Shear Rate γ = 1.3 (1·s^−1^)	Shear Rate γ = 260 (1·s^−1^)
Series I-A	35 vol.% total solid content	5 vol.% of Ni	1320	37.2	1.72	9.65
Series I-B		10 vol.% of Ni	1670	37.7	2.16	9.78
Series I-C		20 vol.% of Ni	1250	27.7	1.62	7.2
Series II-A	50 vol.% total solid content	5 vol.% of Ni	17,900	488	23.3	127
Series II-B		10 vol.% of Ni	22,100	564	28.7	146
Series II-C		20 vol.% of Ni	25,100	498	28.7	156

## Data Availability

Data sharing is not applicable to this article.
